# Hemochromatosis Mutations, Brain Iron Imaging, and Dementia in the UK Biobank Cohort

**DOI:** 10.3233/JAD-201080

**Published:** 2021-02-02

**Authors:** Janice L. Atkins, Luke C. Pilling, Christine J. Heales, Sharon Savage, Chia-Ling Kuo, George A. Kuchel, David C. Steffens, David Melzer

**Affiliations:** a Epidemiology and Public Health Group, University of Exeter Medical School, Exeter, UK; b Medical Imaging, College of Medicine and Health, University of Exeter, Exeter, UK; c Psychology Department, University of Exeter, Exeter, UK and University of Newcastle, Newcastle, NSW, Australia; d Center on Aging, University of Connecticut Health Center, Farmington, CT, USA; e Biostatistics Center, Connecticut Convergence Institute for Translation in Regenerative Engineering, UConn Health, Farmington, CT, USA; f Department of Psychiatry, University of Connecticut Health Center, Farmington, CT, USA

**Keywords:** Cohort, dementia, delirium, gene, hemochromatosis, iron, mutation

## Abstract

**Background:**

Brain iron deposition occurs in dementia. In European ancestry populations, the *HFE* p.C282Y variant can cause iron overload and hemochromatosis, mostly in homozygous males.

**Objective:**

To estimate p.C282Y associations with brain MRI features plus incident dementia diagnoses during follow-up in a large community cohort.

**Methods:**

UK Biobank participants with follow-up hospitalization records (mean 10.5 years). MRI in 206 p.C282Y homozygotes versus 23,349 without variants, including T2^*^ measures (lower values indicating more iron).

**Results:**

European ancestry participants included 2,890 p.C282Y homozygotes. Male p.C282Y homozygotes had lower T2^*^ measures in areas including the putamen, thalamus, and hippocampus, compared to no *HFE* mutations. Incident dementia was more common in p.C282Y homozygous men (Hazard Ratio HR = 1.83; 95% CI 1.23 to 2.72, *p* = 0.003), as was delirium. There were no associations in homozygote women or in heterozygotes.

**Conclusion:**

Studies are needed of whether early iron reduction prevents or slows related brain pathologies in male *HFE* p.C282Y homozygotes.

## INTRODUCTION

Iron has been linked to dementia and other neuro-degenerative diseases, with roles in oxygen transport, myelin production plus synthesis and metabolism of neurotransmitters [1–3]. Several rare genetic ‘Neurodegeneration with Brain Iron Accumulation’ syndromes have been described [4]. Iron accumulation in specific brain areas is found in Alzheimer’s disease (the most common form of dementia), beyond normal accumulations seen in aging [5], and these iron accumulations are associated with amyloid plaques and tau aggregation [5]. Chelator treatment to remove iron has shown promise in Alzheimer’s disease animal models [5] and in human randomized trials, with some evidence of slowing of cognitive decline [6].

The homeostatic iron regulator ‘*HFE*’ gene p.C282Y variant relaxes controls on iron absorption from the gut, leading to moderately raised serum iron levels [7] in heterozygotes. In homozygote ma-les especially, the variants can result in iron overload and iron deposition in many tissues [8]. Male p.C282Y homozygotes are at substantially increased risks of developing clinical hemochromatosis, with related liver cirrhosis and liver carcinomas, arthritis, osteoporosis, and diabetes [9]. Iron overload in p.C282Y homozygote women is less common [7], likely due to iron losses in menstruation. Iron overload can occur but is unusual in p.C282Y and p.C282Y/H63D heterozygotes [9]. In northern European Ancestry populations, the p.C282Y variant is carried by 10–15%, with approximately 1 in 150 (0.67%) being homozygous [10]. In North America, p.C282Y homozygosity prevalence is 0.44% in non-Hispanic whites, but much less common in other ancestry groups [7]. Iron overload in hemochromatosis is prevented and treated with phlebotomy [8], but many patients are currently diagnosed only after irreversible morbidity has developed.

There have been several reports of associations between *HFE* genotypes and dementia, but a re-cent multi-study case-control meta-analysis of the p.C282Y mutation rs1800562 and Alzheimer’s disease reported no association [11]. However, this analysis tested overall genotype associations (which are dominated by the large numbers of heterozygotes) and did not analyze homozygote males separately [11]. It therefore remains unclear whether p.C282Y homozygote men or women have increased risks for adverse brain outcomes.

Given the paucity of evidence, we aimed to test associations between the hemochromatosis (type 1) associated genotypes (*HFE* p.C282Y homozygotes and heterozygotes, plus p.C282Y/H63D status) and; 1) brain features on MRI, and 2) incident dementia recorded during hospitalization. We used data from UK Biobank (UKB) European descent participants (*n* = 335,909), including 28,860 MRI imaging volunteers. We hypothesized that p.C282Y homozygous males would be at most risk from brain iron deposition plus dementia during follow-up, as this group has substantially higher risks of other hemochromatosis morbidities [9]. UKB consent does not allow individual feedback of genotypes, so the medical care and records used in the analyses were not altered by UKB identified genotypes.

## MATERIALS AND METHODS

### Ethical approval

UKB ethical approval was from the North West Multi-Centre Research Ethics Committee. The current analysis was approved under UKB application 14631 (PI David Melzer).

### Data and participants

UKB included 502,634 volunteers aged 40 to 70 years old at recruitment, living near 22 assessment centers in England, Scotland, and Wales [12]. Baseline assessments (2006 to 2010) included disease history [12] and participants consented to genotyping, plus record linkage to the UK National Health Service hospitalization routine datasets.

### Genotypes

As *HFE* p.C282Y mutations are relatively common only in European ancestry groups [7], we used data for European ancestries participants with *HFE* p.C282Y (rs1800562) genotype information, and also for *HFE* p.H63D (rs1799945). UK Biobank used Affymetrix microarrays (800,000 markers dir-ectly genotyped), but as rs1800562 (p.C282Y) was not genotyped, standard imputation methods were applied [13] (see Supplementary Methods for details). Imputed p.C282Y genotypes (rs1800562) were highly correlated with exome sequencing genotypes (from 49,772 participants, r^2^ = 0.998) and only one (of 231, 0.4%) imputed p.C282Y homozyg-otes was incorrectly classified (sequenced genotype = p.C282Y heterozygote). *HFE* p.H63D (rs-1799945) was directly genotyped in the microarray data.

### MRI

Magnetic resonance imaging (MRI) phenotypes were from the UKB brain image-processing pipeline [14]. A standard Siemens Skyra 3T running VD13A SP4, with Siemens 32-channel RF receive head coil was used. Measures included T2^*^ signal loss, an indicator of magnetic susceptibility influenced mainly by iron in deoxyhemoglobin, storage proteins and myelin [15]. Given strong correlations between right and left hemisphere T2^*^ iron deposition measures (correlation 0.48 to 0.79, all *p* < 0.0001), mean values for right and left hemisphere matching variables were used in primary analyses, to reduce multiple statistical testing. However, left and right separate analyses were performed for sensitivity analysis.

Data for MRI analyses were from participants who met the inclusion criteria (scanned May 2014 to 2018), with results of analyses of included participants irrespective of completeness of all derived MRI measures, with additional analysis for those with full data on all derived brain MRI measures.

### Diagnoses and follow-up

Incident diagnoses were from UKB hospitalization routine datasets to March 2020 for England (Hospital Episode Statistics), October 2016 for Scotland (Scottish Morbidity Record), and February 2016 for Wales (Patient Episode Database for Wales). The maximum inpatient follow-up was 14.1 years, mean 10.5 years. Incident diagnoses were ascertained from recorded International Classification of Disease 10th revision codes, including those for dementia (F00^*^; F01^*^; F02^*^; F03^*^; G30^*^); Mild Cognitive Impairment (MCI; ICD-10 code: F06.7); delirium (ICD-10 codes: F05^*^), and for stroke or Transient Ischemic Attack (TIA; ICD-10 codes: G45-G46^*^; I61^*^; I63^*^). Analyses of each incident condition excluded participants with respective prevalent diagnoses at baseline, based on participant responses at baseline interview plus ICD-10 coded hospitaliz-ation records from 1996 to baseline interview (see Supplementary Methods). For example, participants reporting doctor diagnosed dementia at baseline interview and those with dementia in hospitalization data before baseline interview were excluded from incident dementia analyses. Dementia diagnosis accuracy in English hospital records has been validated (sensitivity 78%, specificity 92%) [16] and Scottish routine data (positive predictive value 87%) [17]. In our preparatory analyses, Apolipoprotein E (*APOE*) *ɛ*4/*ɛ*4 type was strongly associated with the recorded incident dementia diagnoses (OR = 7.8 95% CI: 7.0 to 8.6, versus *ɛ*3/*ɛ*3), as expected.

### Statistical analysis

All UK Biobank participants of European genetic ancestry with the hemochromatosis (type 1) related genotype data were included in analyses: i.e., *HFE* p.C282Y homozygotes and (simple) heterozygotes, plus p.C282Y/H63D compound heterozygotes, plus those with neither variant as a comparison group. Participants with the H63D heterozygote and hom-ozygote variant only were excluded, as these genotypes are not risk factors for hemochromatosis. Given that *HFE* p.C282Y homozygotes (mainly men) and to a lesser extent the p.C282Y/H63D genotype are at most risk for iron overload, analyses and reporting focusses on these genotypes. See Supplementary Methods for details and Supplementary Table 1 for participant numbers for the studied genotype groups.

We excluded the relatively small number of participants without imputed genotypes (*n* = 15,233/502,642, 3.0%) and those with imprecise p.C282Y im-putation (*n* = 183/487,409, 0.04%). Also, participants who were p.H63D homozygotes (*n* = 10,258) or p.H63D heterozygotes (*n* = 105,019) only were excluded from diagnosis analyses, and from the MRI sub-study (*n* = 802 and *n* = 8,976 respectively), as this genotype causes modestly higher blood iron levels but is not associated with iron overload. MRI measures were quantile normalized and z-transformed before analysis. For MRI analyses, we hypothesi-zed that T2^*^ iron deposition associated measures were most likely to be associated with *HFE* genotypes, followed by gray matter volumes. However, to be conservative and complete, we analyzed all available UK Biobank produced MRI measures, with Benjamini-Hochberg adjustment for multiple testing applied across all imaging phenotype associations (*n* = 481 tests in p.C282Y homozygotes), to limit the false discovery rate at 5%. Linear regression was used to test associations between *HFE* genotypes (p.C282Y homozygotes, p.C282Y/H63D compound heterozygotes, and p.C282Y heterozygotes) and MRI measures (after calculating mean left and right measures as above), compared with having neither mutations, in males and females separately.

Cox proportional hazards regression was used to test genotype associations with incident diagnoses. All models were stratified by sex and adjusted for population genetics sub-structure using the first ten principal components generated in European-descent participants, genotyping microarray (Affymetrix Axiom array for 90% and Affymetrix BiLEVE array for 10% of participants, sharing > 95% content), assessment center, plus age at baseline assessment. The model estimating associations between genotype and incident dementia was also adjusted for *APOE* genotype, and the interaction between the genotype and *APOE* *ɛ*4 specifically was tested for, since previous studies have suggested an interaction between the HFE gene and *APOE* *ɛ*4 in conferring risk for Alzheimer’s disease [18, 19]. There were no violations of proportional hazards assumptions for our principal outcome (dementia) in Cox models. Analyses used Stata v15.1, and ‘stcox’ function for Cox models. In sensitivity analyses, we also excluded one randomly selected participant from each pair of participants related to the third degree (*n* = 53,376) leaving 282,533 unrelated participants, to avoid possible inflation of associations from family relatedness. In an additional sensitivity analysis focused on older subjects who are more likely to develop dementia, we excluded participants aged under 60 years (*n* = 186,422) leaving 149,487 participants. A further sensitivity analysis excluded participants with hemochromatosis diagnosed at baseline (*n* = 321), to provide separate estimates for community identified undiagnosed participants (*n* = 335,588).

Missing data: UK Biobank derived MRI gray matter volume measures were available for slightly more participants than for T2^*^ measures: we therefore provide analyses of all participant data for each brain MRI measure, and additional analyses including only participants with a full set of MRI measures. Diagnosis data during follow-up were from all available hospital inpatient records.

## RESULTS

### MRI measure associations

Data from the UK Biobank brain MRI sub-study were available (Table 1) including 78 male and 128 female p.C282Y homozygotes, plus 11,082 male and 12,257 female participants with neither *HFE* variant. Overall participation in brain MRI was slightly more common in males (OR = 1.06 95% CI 1.04 to 1.09) and slightly less common with advancing age (OR = 0.970 CI 0.969 to 0.971 per year increase in age). However, male p.C282Y homozygotes in UK Biobank were less likely to participate in the MRI sub-study (5.95% of 1,294 homozygote males versus 8.94% of men with neither p.C282Y nor p.H63D mutations: OR = 0.68 95% CI 0.54 to 0.85). Eleven male p.C282Y homozygote MRI participants (of the 78, 14.1%) were diagnosed with hemochromatosis at UK Biobank baseline, compared to 12.1% in the 1,294 homozygote males included in diagnosis analyses. Female p.C282Y homozygote participation in the MRI subset was similar to participation with neither *HFE* mutation.

**Table 1:** Characteristics of participants included in the imaging and disease analyses, by HFE genotype and sex

Characteristics of participants	Male			Female		
	Neither *HFE*variant	C282Y+/H63D+	C282Y+/+	Neither *HFE*variant	C282Y+/H63D+	C282Y+/+
**MRI subgroup**
Total number	11,082	468	78	12,257	453	128
Age –mean (sd)	64.2 (7.7)	64.3 (7.9)	63.1 (8.3)	63.0 (7.4)	63.2 (7.3)	64.7 (7.5)
**Diagnosis study**
Number of participants	122,860	4,955	1,294	145,719	5,746	1,596
Age (baseline) –mean (sd)	57.0 (8.1)	56.9 (8.1)	56.8 (8.2)	56.6 (7.9)	56.5 (7.9)	56.9 (8.0)
Hemochromatosis diagnosis (baseline)	30	29	156	8	12	54
Incident diagnoses^*^
Dementia	1,358	64	25	1,164	47	16
MCI	210	9	3	147	9	3
Delirium	1,313	61	24	932	38	14
Stroke/TIA	2,895	131	39	2,148	81	17

^*^Excluding diagnoses at baseline: some participants had more than one diagnosis. See Supplementary Table 1 for other genotype groups.

**Fig. 1:**
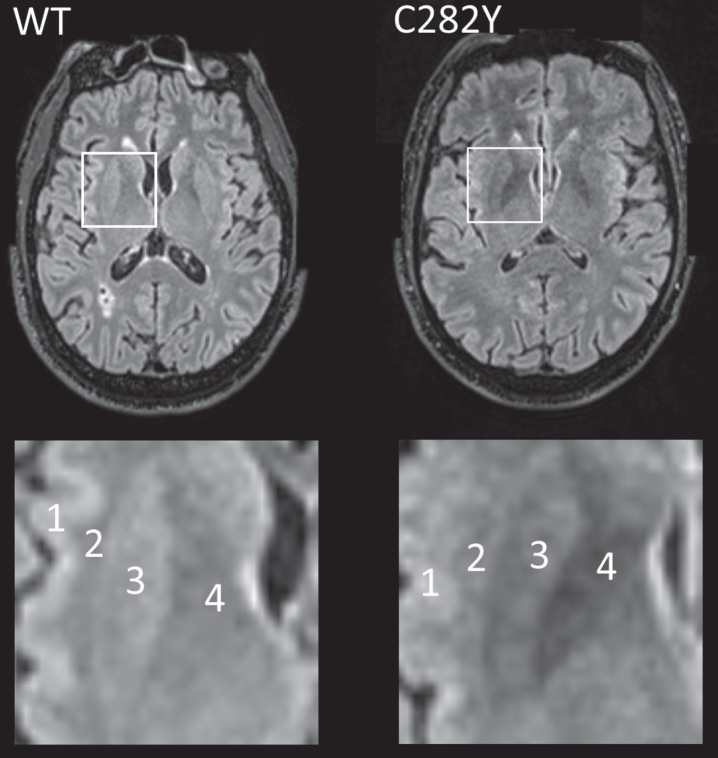
MRI T2 FLAIR axial images for male participants closest to respective mean putamen T2^*^ values with p.C282Y homozygote variants (right) and neither *HFE* variant (left, ‘WT’). In the highlighted square, cortical (1) and white matter (2) intensity appears similar in both images, but the p.C282Y homozygote image shows relative hypo-intensity (associated with iron deposition) in the putamen (3) and globus pallidus (4). Images provided by UK Biobank copyright under license. WT, wild type or neither p.C282Y nor p.H63D mutations present. See details of image acquisition in Supplementary Material.

Data on T2^*^ signal loss (lower measures associated with more iron deposition) were available for 78 male and 128 female homozygotes (Table 2). T2^*^ measures were substantially lower in male p.C282Y homozygotes compared to those with neither variants across a number of brain regions including the putamen, caudate, and nucleus accumbens, but notably also within important cognitive regions: the thalamus (beta = –0.90 standard deviations ‘sd’, 95% CI –1.12 to –0.68, Benjamini-Hochberg multiple testing adjusted *p*-value = 1.4^*^10^–13^) and hippocampus (beta = –0.32 sd, 95% CI –0.52 to –0.13, adjusted *p* = 0.022). For illustration (Fig. 1), we present T2 fluid attenuated inversion recovery (FLAIR) images from a male homozygote with the nearest to group T2^*^ putamen measure, versus a similar image from an age and sex-matched participant without *HFE* mutations.

**Table 2:** Brain MRI median T2^*^ and gray matter volume associations from linear regressions, comparing HFE p.C282Y homozygotes to those without p.C282Y or p.H63D variants), in males and females separately

Outcome description	beta (standard deviations)	95% CI (lower)	95% CI (upper)	*p*	multiple testing adjusted
**Males**
Median T2^*^ measures (72 HFE p.C282Y homozygotes versus 9,304 controls)
Putamen	–0.91	–1.13	–0.70	1.0^*^10–16	4.9^*^10–14
Thalamus	–0.90	–1.12	–0.68	5.7^*^10–16	1.4^*^10–13
Caudate	–0.55	–0.79	–0.32	3.4^*^10–6	2.1^*^10-4
Nucleus Accumbens	–0.40	–0.61	–0.19	2.2^*^10–4	6.6^*^10-3
Pallidum	–0.33	–0.52	–0.13	8.6^*^10-4	2.2^*^10-2
Hippocampus	–0.32	–0.52	–0.13	9.2^*^10-4	2.2^*^10-2
*Gray matter volume (77 HFE p.C282Y homozygotes versus 10,364 controls)*
Putamen	–0.86	–1.08	–0.64	1.1^*^10–14	1.8^*^10–12
Ventral Striatum	–0.71	–0.92	–0.50	8.0^*^10-11	9.8^*^10-9
Vermis IX Cerebellum	–0.80	–1.04	–0.55	1.7^*^10-10	1.6^*^10-8
V Cerebellum	–0.66	–0.89	–0.42	4.3^*^10-8	3.5^*^10-6
I-IV Cerebellum	–0.57	–0.81	–0.33	2.7^*^10-6	1.9^*^10-4
Vermis VIIIb Cerebellum	–0.49	–0.71	–0.27	1.3^*^10-5	6.4^*^10-4
Vermis VIIb Cerebellum	–0.50	–0.75	–0.25	9.7^*^10-5	3.6^*^10-3
Vermis X Cerebellum	–0.54	–0.81	–0.27	9.0^*^10-5	3.6^*^10-3
Vermis VIIIa Cerebellum	–0.46	–0.70	–0.22	1.9^*^10-4	6.4^*^10-3
Caudate	–0.37	–0.60	–0.14	1.8^*^10-3	3.8^*^10-2
Vermis VI Cerebellum	–0.38	–0.62	–0.13	2.3^*^10-3	4.2^*^10-2
**Females**
Median T2^*^ measures (113 HFE p.C282Y homozygotes versus 10,491 controls)
Putamen	–0.88	–1.07	–0.69	5.3^*^10–19	2.6^*^10–16
Thalamus	–0.70	–0.87	–0.53	1.6^*^10–15	2.3^*^10–13
Caudate	–0.57	–0.77	–0.37	2.0^*^10-8	1.2^*^10-6
Pallidum	–0.41	–0.59	–0.24	4.4^*^10-6	1.8^*^10-4
*Gray matter volume (128 HFE p.C282Y homozygotes versus 11,537 controls)*
Putamen	–0.78	–0.96	–0.59	5.5^*^10–16	1.3^*^10–13
Vermis IX Cerebellum	–0.74	–0.92	–0.56	1.9^*^10–15	2.3^*^10–13
Ventral Striatum	–0.62	–0.78	–0.45	6.6^*^10–14	6.4^*^10–12
Vermis VIIIa Cerebellum	–0.53	–0.70	–0.36	6.7^*^10-10	4.7^*^10-8
V Cerebellum	–0.47	–0.64	–0.29	1.0^*^10-7	5.6^*^10-6
Vermis VIIb Cerebellum	–0.42	–0.59	–0.25	2.0^*^10-6	9.6^*^10-5
I-IV Cerebellum	–0.37	–0.52	–0.21	4.2^*^10-6	1.8^*^10-4
Vermis VI Cerebellum	–0.40	–0.58	–0.23	4.8^*^10-6	1.8^*^10-4
Vermis VIIIb Cerebellum	–0.40	–0.57	–0.23	5.9^*^10-6	2.0^*^10-4
Caudate	–0.42	–0.61	–0.22	2.2^*^10-5	6.7^*^10-4
Vermis X Cerebellum	–0.30	–0.47	–0.13	4.5^*^10-4	1.1^*^10-2
Thalamus	–0.29	–0.47	–0.11	1.7^*^10-3	3.2^*^10-2
Vermis Crus II Cerebellum	–0.26	–0.43	–0.09	2.8^*^10-3	4.8^*^10-2

Linear regression models for mean of left and right measures, adjusted for age, imaging assessment center, microarray, and genetic principal components 1–10. Beta, standard deviation difference in MRI imaging phenotype between C282Y homozygous group and neither p.C282Y nor p.H63D variants. CIs, confidence intervals. Adjusted p, Benjamini-Hochberg adjusted *p*-values for 481 tests. See Supplementary Tables 2, 3 for full results with other genotype groups. See Supplementary Tables 4, 5 for analysis of left and right hemispheres separately.

Smaller gray matter volumes were found in the putamen of male C282Y homozygotes (beta –0.86 sd, 95% CI –1.08 to –0.64, adjusted *p* = 1.8^*^10^–12^), caudate, several cerebellar regions, and the ventral striatum, compared to neither variants (see Supplementary Tables 2, 3 for full results). Results of left and right hemisphere measures analyzed separately were similar (Supplementary Tables 4, 5). In a sensitivity analysis including participants with complete MRI measures only (Supplementary Tables 6, 7) results were also similar to those presented above.

In women, there were also associations between p.C282Y homozygote status and T2^*^ measures within the putamen, thalamus, caudate, and pallidum (Table 2). Lower gray matter volumes were observed in the putamen, caudate, thalamus, ventral striatum, and regions of the cerebellum (adjusted *p* < 0.05). In male and female C282Y/H63D heterozygotes and in the other *HFE* genotype groups there were similar associations, but effect sizes were substantially smaller (≤0.5 standard deviations - see Supplementary Tables 2, 3).

### Disease associations

Analyses of incident diagnoses during follow-up included UKB participants of European descent, aged 40 to 70 years at baseline (mean 56.8 years, SD 8.0) (Table 1). Only 12.1% (156/1,294) of male and 3.4% (54/1,596) of female p.C282Y homozygotes had been diagnosed with hemochromatosis (Table 1) at UK Biobank baseline interview. (See Supplementary Table 1 for details of studied *HFE* genotypes including p.C282Y heterozygotes).

**Fig. 2:**
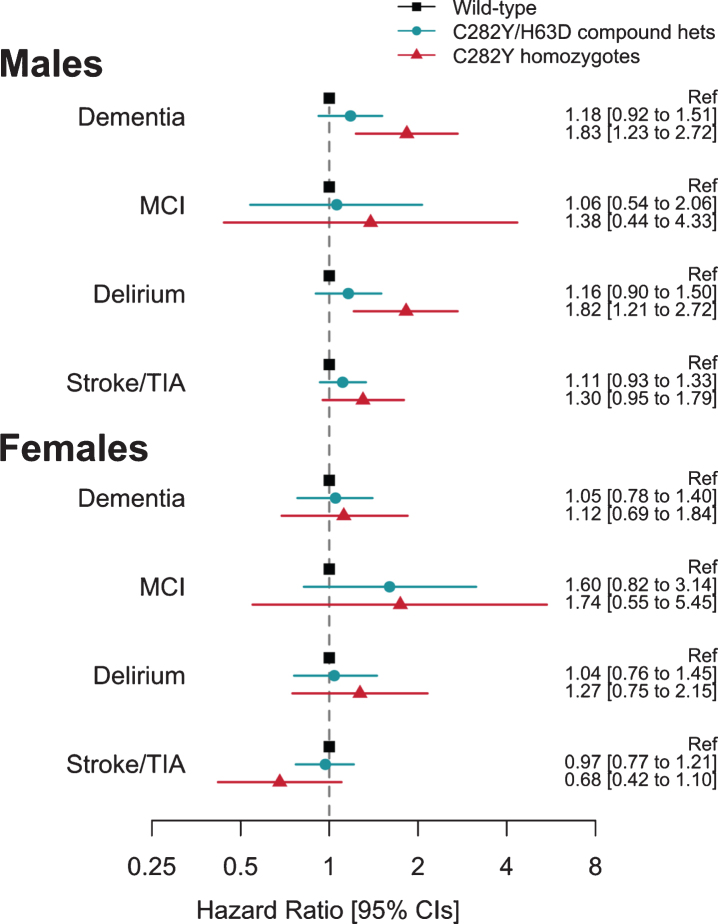
Association between hemochromatosis risk HFE genotypes and incident diagnoses: Hazard ratios (95% Confidence intervals) compared to those with neither HFE mutation. Cox proportional hazards regression adjusted for age, imaging assessment center, microarray, and genetic principal components 1–10. Excluding each respective diagnosis at baseline. Full results for heterozygotes are presented in Supplementary Table 8.

During follow-up, 25 male homozygotes (16 fe-male) and 1,358 males without *HFE* variants were diagnosed with incident dementia (Table 1; Supplementary Table 1). Male p.C282Y homozygotes had increased hazards for incident dementia (HR 1.83, 95% CI 1.23 to 2.72, *p* = 0.003, compared to those with neither p.C282Y nor p.H63D) (Fig. 2; Supplementary Table 8). This association was similar after additionally adjusting for APOE genotype status (HR 1.83, 95% CI 1.23 to 2.73, *p* = 0.003) and there was no statistically signi-ficant interaction between any p.C282Y/H63D genotype and *APOE* *ɛ*4 (*p* > 0.05). To remove potential bias from multiple family members, in a sensitivity analysis we included only one participant from those related to the third degree or closer (*n* = 130,275 after exclusions): the male homozygous association with dementia was similar (HR = 1.88, 95% CI: 1.22 to 2.90, *p* = 0.004). To focus on dementia onsets in later life only, we included only participants aged 60 years or above at baseline in a sensitivity analysis (*n* = 70,710 after exclusions): the male homozygous association with incident dementia was relatively un-changed (HR = 1.73, 95% CI: 1.13 to 2.67, *p* = 0.01; Supplementary Table 9). The association between male p.C282Y homozygosity and incident dementia remained significant after excluding 242 participa-nts with hemochromatosis diagnosed at baseline from the total sample (*n* =153,446) (HR: 1.81, 95% CI: 1.19 to 2.76, *p* = 0.01; Supplementary Table 10).

To extend the evidence of association between p.C282Y homozygosity and dementia in men, we add-itionally tested associations with mild cognitive im-pairment, delirium, and stroke or transient ischemic attack diagnoses. There were increased hazards for diagnosis of incident delirium in p.C282Y homozygote males (HR 1.82, CI 1.21 to 2.72, *p* =0.004), but no association with mild cognitive impairment, or stroke and transient ischemic attack.

In female C282Y homozygotes and the heterozygote *HFE* variants in either sex, there were no associations with dementia or the other studied diagnoses (Fig. 2) (see Supplementary Table 8).

## DISCUSSION

We aimed to estimate hemochromatosis genotype associations with brain MRI measures and incident dementia in the UKB community genotyped cohort, with a specific focus on male *HFE* p.C282Y homozygotes, who develop the majority of hemochromatosis related physical diseases. We found associations in p.C282Y homozygote men with lower T2^*^ measures (associated with greater iron deposition [15]) in several areas involved in dementia, including the hippocampus and thalamus. In addition, we found lower gray matter volumes including in the putamen and ventral striatum. In the main UK Biobank sample (*n* = 335,909), we found a substantial increase in hazards for incident dementia diagnoses during hospitalization in p.C282Y homozygote men HR = 1.83 95% CI 1.23 to 2.72) during the mean 10.5 year follow-up, plus increased hazards for delirium, for which dementia is a major risk factor [20]. Overall, these results suggest that p.C282Y homozygosity is a significant risk factor for dementia in men with European ancestries. Associations between p.C282Y heterozygous status and dementia were not statistically significant.

It is difficult to compare our findings with similar community studies with sufficient numbers of p.C282Y homozygote men or women, because UKB contains nearly 10 times more such participants than the previously largest similar study [9], yielding far more statistical power to detect associations. As noted, iron deposition has been linked to core Alzheimer’s disease pathologies, with iron being bound by Alzheimer’s associated amyloid-β and tau, and also involved in formation of oligomeric tau [2]. A recent meta-analysis of the p.C282Y variant (HFE rs1800562, plus the transferrin variant rs1049296) found no association with Alzheimer’s disease, but no results were provided on the homozygote mutation in either men or women. In UK Biobank, a similar analysis of rs1800562 also produces no overall association with incident dementia, but this is because there were 64,458 heterozygotes (Supplementary Table 1) and only 2,890 homozygotes in analyses. An earlier meta-analysis [21] of p.C282Y dementia association studies included data on only 18 p.C282Y homozygote cases and 37 controls and reported no association, while another meta-analysis [22] studied 16 p.C282Y heterozygotes and reported reduced Alzheimer’s disease risks, but included no data on homozygotes.

The brain areas with associated iron deposition in p.C282Y homozygote men included the hippocampus and thalamus, two structures known to play important roles in memory function [23], and which are frequently affected in early Alzheimer’s disease [24], as well as in the putamen, where iron accumulation occurs with aging [25] and in geriatric depression [26]. Smaller putamen gray matter volumes have been reported in neurodegenerative disorders [27], including Alzheimer’s disease [28].

De Barros et al. [29] recently reviewed the evidence on correlations between MRI relaxation measures and direct physical measures (including mass spectrometry) of iron in brain samples, and reported overall high linear correlations. These correlations were strong in the basal ganglia (Pearson correlation coefficient *r* = 0.90) and grey matter, but there was little correlation in white matter, which Langkammer et al. [30] postulated might be due to the effects of paramagnetic iron and diamagnetic myelin. The higher iron versus MRI relaxation correlations in the basal ganglia and grey matter is consistent with our reported C282Y associations being concentrated in these areas.

### Limitations

The main limitation of this analysis is that dementia sub-type data are limited in UKB hospitalization records: within the male p.C282Y homozygotes with dementia, specific diagnoses were Alzheimer’s disease (seven participants, ICD-10 F00 & ICD-10 G30); vascular dementia (four participants, ICD-10 F01); dementia in other specified diseases classified elsewhere (three participants, ICD-10 F02); and unspecified dementia (*n* = 11, ICD-10 F03). More work is therefore needed to systematically assess specific dementia pathologies, including in those not hospitalized. Unfortunately, there is currently little known about the molecular localization of excess iron in the brains of patients with hemochromatosis, e.g., whether the iron is present as hemosiderin deposition, in some ferritin bound form, or in some other form. Also, UKB volunteers tended to have somewhat less morbidity and lower prevalence of health risk factors at baseline compared to the UK population [31], but this should have limited impact on our estimates, which are based on incident diagnoses during follow-up only in those free of dementia diagnoses at baseline. In female p.C282Y homozygotes and in heterozygotes, brain iron deposition appeared less marked, but longer follow-up will be needed to exclude associations with dementia.

These findings add to the extensive excess physical morbidity reported in p.C282Y homozygote males, including excess liver disease, arthritis, diabetes, osteoporosis, and pneumonia [9]. More work is needed to confirm that the excess dementia and delirium seen in male p.C282Y homozygotes is causally related to the iron deposition directly rather than by a secondary mechanism, for example as a consequence of co-existing liver cirrhosis. However, in our study sample, only one p.C282Y homozygote male with dementia had also been diagnosed with liver cirrhosis or fibrosis. Iron excess is usually easily and safely treated with venesection [8], and therefore early diagnosis and treatment of *HFE* p.C282Y homozygote men may results in lower rates of dementia and delirium, whether these conditions are due to excess iron directly or from a secondary causal mechanism. Clinical trials may also be needed to establish whether dementia in this group responds to iron reduction therapy.

In summary, men with the *HFE* p.C282Y homozygous mutation developed substantially more marked brain iron deposition in dementia relevant brain areas. In addition, p.C282Y homozygote men were more likely to be diagnosed with dementia during a 10.5 year mean follow-up in hospitalization data. Studies are needed of whether early ascertainment of hemochromatosis and intervention in *HFE* p.C282Y homozygotes may prevent or limit associated dementia related brain pathologies.

## DATA SHARING

Data are available on application to the UK Biobank (www.ukbiobank.ac.uk/register-apply).

## Supplementary Material

Supplementary Material
