# Supplementary material for: Hemochromatosis Mutations, Brain Iron Imaging, and Dementia in the UK Biobank Cohort
Source: J Alzheimers Dis. 2021 Feb 2;79(3):1203–11. doi: 10.3233/JAD-201080 (PMC7990419; doi:10.3233/JAD-201080)
Supplement: Supplementary Material [file jad-79-jad201080-s001.pdf]

Supplementary Material

Hemochromatosis Mutations, Brain Iron Imaging, and Dementia in the UK Biobank Cohort

Supplementary Methods

Genotyping

Genetic data were available on 488,377 UK Biobank participants after genotype calling and quality control performed centrally by the UK Biobank team [1], and in this analysis we used data from the v3 imputed genotype release (released 2018). In brief, 1000 Genomes phase 3 data was used as the reference panel for phasing of the autosomes (using SHAPEIT3), followed by imputation of genetic variants using first the Haplotype Reference Consortium panel, and secondly combined data from UK10K and 1000 Genomes phase 3 panels (using IMPUTE4): this resulted in 93,095,623 autosomal genetic variants (including single nucleotide polymorphisms, indels, and some larger structural variants) in 487,442 participants.

We used 451,427 participants identified as 'white European' through self-report and verified through principal components (PC) analysis based on genotypes (described in detail by Thompson et al. [2]). Briefly, PCs were generated in the 1000 Genomes Cohort using high-confidence genetic variants to obtain their individual loadings. These loadings were used to project UK Biobank participants into the same PC space. PCs 1 to 4 were used to identify participants of European descent. Related individuals were identified through kinship analysis using the KING software [3].

C282Y is a single nucleotide polymorphism (SNP) on chromosome 6 (b37 position 26093141, rsID rs1800562) in the *HFE* gene. H63D is a SNP also in the *HFE* gene (b37 position 26091179, rsID rs1799945). Both are missense variants causing a single amino-acid change in HFE. In the UK Biobank participants, the SNPs were not correlated (R^2=0.015). rs1800562 was not directly genotyped, therefore imputed C282Y genotypes were used (data

from the 2018 v3 release): 445,521 participants (98.7% of 451,427) were imputed with 100% confidence and 5,723 were recoded (i.e., estimated genotype dose between 0 and 0.25 set to 0, values between 0.75 and 1.25 set to 1, and finally between 1.75 and 2 to 2); 183 participants (0.04%) were excluded due to imprecise imputation, yielding 451,243 participants in analyses. A small number of participants have since withdrawn from the UK Biobank, leaving the final analysis set of 451,186 for analyses.

Disease ascertainment

Disease ascertainment at baseline was by self-reported doctor diagnoses (verified by a trained nurse during the verbal interview), plus diagnoses recorded in linked ICD10 coded inpatient hospital records covering the period 1996 to the date of participant baseline interview. Incident hospital admissions data were from Hospital Episode Statistics (HES) for England, the Patient Episode Database for Wales (PEDW) and the Scottish Morbidity Record (SMR) [4]. National death certification was also used. In analysis of incident diagnoses based on HES, the date of censoring for each participant was either March 2020 (for participants in England), October 2016 (for participants with Scotland), or February 2016 (for participants in Wales). If the participant died, then this date was used instead.

Selection and analysis of participants for MRI figure

Two UK Biobank participants were chosen to illustrate the differences between C282Y homozygotes and those without any HFE mutations. In the male C282Y homozygous population with complete MRI data in the first 20,000 data release (n=34 male C282Y homozygotes), six were excluded due to having a diagnosis of haemochromatosis (prevalent or incident, either self-reported, from HES, or from the primary care data, where available). The remaining participants (n=28) had no recorded diagnoses of dementia, mild cognitive

impairment, Parkinson’s disease, or strokes and were therefore eligible for inclusion. We selected the participant closest to the mean T2* putamen for that group. We then selected a participant of the same age (60 years) with no *HFE* C282Y or H63D genotypes (and no haemochromatosis, dementia, mild cognitive impairment, Parkinson’s disease, or stroke) who was closest to the mean T2* putamen for this control group.

Sequences acquired included T1-weighted structural images, susceptibility weighted images (SWI) and T2-weighted FLAIR images. Whilst the SWI sequences are most sensitive to iron deposition, and has been utilised for quantitative analysis, the image resolution is such that the anatomy is not clearly demonstrated. Therefore, we examined the T2-FLAIR images to provide an example of signal changes seen in the putamen identified in the analysis of T2* SWI parameters and C282Y genotype. The T2-FLAIR images were acquired in the sagittal plane and reconstructed axially. See the UK Biobank brain MRI documentation for more detail regarding image generation and processing, including “de-facing” for anonymization (http://biobank.ctsu.ox.ac.uk/showcase/showcase/docs/brain_mri.pdf).

T2 FLAIR images in NIFTI format were examined by a diagnostic radiographer (Dr Christine J Heales, who has further qualifications in MRI reporting of the head and spine) using the MRIcron (http://www.mricro.com) software. The software used did not enable the baseline to be adjusted so the axial slices were anatomically matched for both the control and case example as closely as possible using the corpus callosum and the lateral ventricles as reference points: see Supplementary Figure 1.

Supplementary Figure 1. Selection of the axial slice of the T2 FLAIR MR images

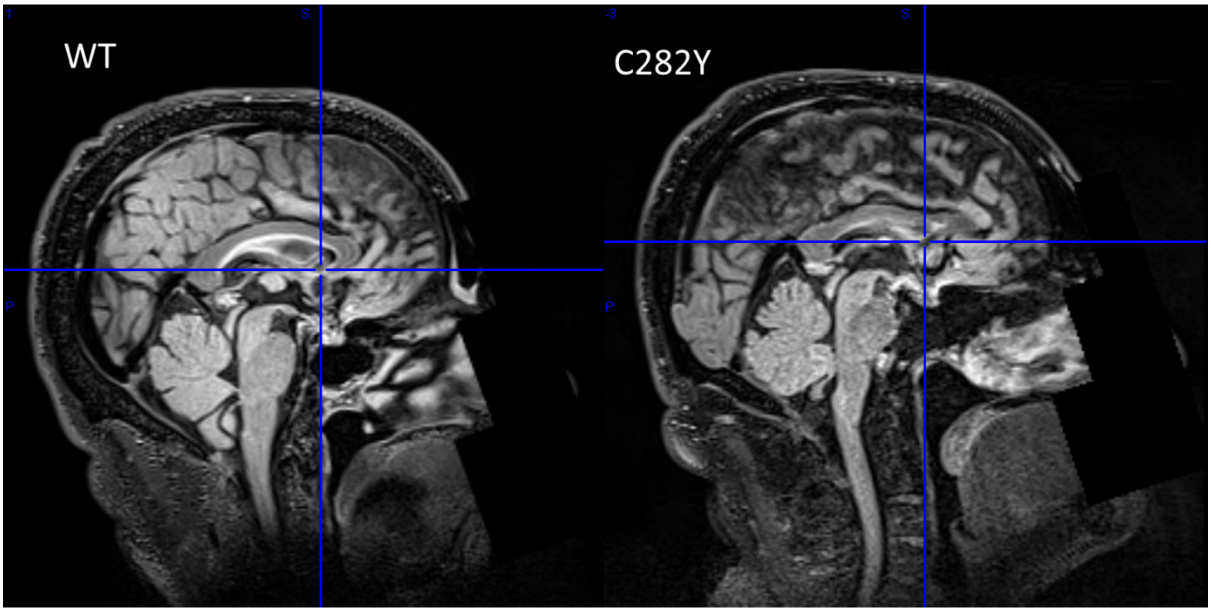

Horizontal blue line shows the axial slice from which the putamen comparison was made. Images provided by UK Biobank © under licence.

Supplementary Tables

Due to the size of some of the tables these are stored in the cloud and can be accessed here:

https://www.dropbox.com/s/s9rftj1s2wjey4l/Melzer%202020%20HFE%20Brain%20-%20Supplementary%20Tables%2020Jul2020.xlsx?dl=0

REFERENCES

[1] Bycroft C, Freeman C, Petkova D, Band G, Elliott LT, Sharp K, Motyer A, Vukcevic D, Delaneau O, O'Connell J, Cortes A, Welsh S, Young A, Effingham M, McVean G, Leslie S, Allen N, Donnelly P, Marchini J (2018) The UK Biobank resource with deep phenotyping and genomic data. *Nature* **562**, 203–209.

[2] Thompson WD, Tyrrell J, Borges M-C, Beaumont RN, Knight BA, Wood AR, Ring SM, Hattersley AT, Freathy RM, Lawlor DA (2019) Association of maternal circulating 25(OH)D and calcium with birth weight: A mendelian randomisation analysis. *PLoS Med* **16**, e1002828.

[3] Manichaikul A, Mychaleckyj JC, Rich SS, Daly K, Sale M, Chen W-M (2010) Robust relationship inference in genome-wide association studies. *Bioinformatics* **26**, 2867–2873.

[4] Sudlow C, Gallacher J, Allen N, Beral V, Burton P, Danesh J, Downey P, Elliott P, Green J, Landray M, Liu B, Matthews P, Ong G, Pell J, Silman A, Young A, Sprosen T, Peakman T, Collins R (2015) UK Biobank: An open access resource for identifying the causes of a wide range of complex diseases of middle and old age. *PLoS Med* **12**, e1001779.
